# The *N*-Reductive System Composed of Mitochondrial Amidoxime Reducing Component (mARC), Cytochrome b5 (CYB5B) and Cytochrome b5 Reductase (CYB5R) Is Regulated by Fasting and High Fat Diet in Mice

**DOI:** 10.1371/journal.pone.0105371

**Published:** 2014-08-21

**Authors:** Heyka H. Jakobs, Michal Mikula, Antje Havemeyer, Adriana Strzalkowska, Monika Borowa-Chmielak, Artur Dzwonek, Marta Gajewska, Ewa E. Hennig, Jerzy Ostrowski, Bernd Clement

**Affiliations:** 1 Department of Pharmaceutical and Medicinal Chemistry, Christian-Albrechts-Universität zu Kiel, Kiel, Germany; 2 Department of Genetics, Maria Sklodowska-Curie Memorial Cancer Center and Institute of Oncology, Warsaw, Poland; 3 Department of Gastroenterology and Hepatology, Medical Center for Postgraduate Education, Warsaw, Poland; IRCCS Istituto Oncologico Giovanni Paolo II, Italy

## Abstract

The mitochondrial amidoxime reducing component mARC is the fourth mammalian molybdenum enzyme. The protein is capable of reducing *N*-oxygenated structures, but requires cytochrome b5 and cytochrome b5 reductase for electron transfer to catalyze such reactions. It is well accepted that the enzyme is involved in *N*-reductive drug metabolism such as the activation of amidoxime prodrugs. However, the endogenous function of the protein is not fully understood. Among other functions, an involvement in lipogenesis is discussed. To study the potential involvement of the protein in energy metabolism, we tested whether the mARC protein and its partners are regulated due to fasting and high fat diet in mice. We used qRT-PCR for expression studies, Western Blot analysis to study protein levels and an *N-*reductive biotransformation assay to gain activity data. Indeed all proteins of the *N*-reductive system are regulated by fasting and its activity decreases. To study the potential impact of these changes on prodrug activation *in vivo*, another mice experiment was conducted. Model compound benzamidoxime was injected to mice that underwent fasting and the resulting metabolite of the *N*-reductive reaction, benzamidine, was determined. Albeit altered *in vitro* activity, no changes in the metabolite concentration *in vivo* were detectable and we can dispel concerns that fasting alters prodrug activation in animal models. With respect to high fat diet, changes in the mARC proteins occur that result in increased *N*-reductive activity. With this study we provide further evidence that the endogenous function of the mARC protein is linked with lipid metabolism.

## Introduction

**Figure 1:**
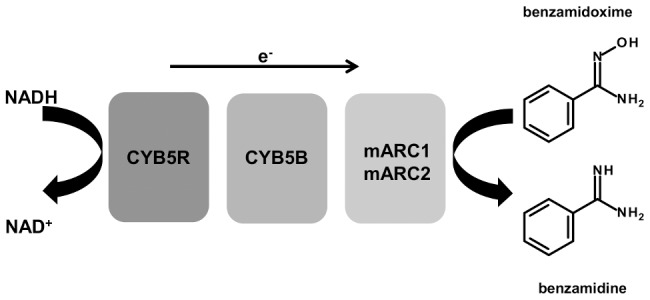
Scheme of the *N*-reductive reaction with model compound benzamidoxime by mARC, CYB5B and CYB5R. Electrons are transferred from NADH via FADH in CYB5R, hem in CYB5B and Moco in mARC1/2 to benzamidoxime, the latter is reduced to benzamidine.

The mitochondrial amidoxime reducing component (mARC) is the fourth mammalian molybdenum containing enzyme besides sulfite oxidase, xanthine oxidase and aldehyde oxidase [1], [2]. All so far fully sequenced and annotated mammalian genomes encode two isoforms mARC1 and mARC2, which show a high degree of sequence similarities [3]. Both enzymes are capable of reducing *N*-oxygenated structures, but require at least mitochondrial cytochrome b_5_ (CYB5B) [4], [5] in cell culture or both CYB5B and NADH-cytochrome b_5_ reductase (CYB5R) [5]–[7] for activity reconstitution *in vitro* (Figure 1). The activity of the three component system depends on the molybdenum cofactor bound to mARC and hem in CYB5B [3], [5], [6]. It is well accepted that the enzyme plays a major role in *N*-reductive drug metabolism and is thus a counterpart of the main drug oxidizing enzymes cytochrome P450s (P450) and flavin-containing monooxygenases (FMO). The *N*-reductive reaction of mARC and its partners is likewise involved in the activation of amidoxime prodrugs, precursor molecules of active drugs with improved pharmacokinetic properties [8]–[10].

However, little is known about the endogenous functions of the enzyme system and further studies on this topic are necessary. An involvement in detoxification, as common for drug metabolizing enzymes, was proven: The enzyme is capable of detoxifying hydroxylated and mutagenic DNA-bases [7] and mARC2 was shown to be downregulated in colorectal carcinoma tissues [11].

Nevertheless, the involvement in other metabolic pathways is also discussed, including NO metabolism where the enzyme is involved in the reduction of NO precursor substrate *N* ^ω^-hydroxy-L-arginine [6] and furthermore capable of reducing nitrite to NO under anaerobic conditions [12].

Moreover, increased mRNA levels for mARC2 have been found in diabetic animal models and in cell culture under high glucose conditions [13]. High abundances of the enzyme after differentiation of 3T3-cells to adipocytes were shown [4], [14] and recently Neve *et al.* provided evidence on its function in lipid synthesis [4].

As the latter findings indicate an involvement in energy metabolism, we studied how changes in energy supply influence the mARC containing enzyme system. Besides cell culture experiments, we studied changes in mice under fasting conditions, high fat diet (HFD) and in leptin receptor-deficient mice. Under the hypothesis that the enzyme system is connected with energy metabolism, changes of the mARC proteins and its partners are assumable in these states. Fasting in mice causes body weight loss, as well as changes in cardiovascular, hormonal and metabolic parameters and hepatic metabolism [15]. In contrast, HFD and leptin-receptor-deficiency results in an obese habitus with most of the symptoms of the metabolic syndrome [16]. In both cell culture and mice experiments, we focused on mRNA and protein levels such as the *in vitro* activity of the enzyme complex. Indeed, changes in the three components of the enzyme system due to glucose in cell culture and due to fasting and HFD in mice were detectable and support the assumption that the enzyme system is connected with energy metabolism. Additionally, we tested whether food deprivation in mice influences *N*-reductive metabolite concentrations *in vivo*. Potential changes in *N*-reductive activity due to fasting could imply problems in both preclinical drug testing in rodents and in the application of mARC-activated prodrugs. However, we have been able to dispel these concerns during this study.

## Materials and Methods

### Materials

Methanol HPLC grade was purchased from J. T. Baker (Deventer, The Netherlands). PBS was from PAA Laboratories GmbH (Pasching, Austria). FBS and DMEM without glucose were from gibco (Life Technologies, Carlsbad, USA). Other chemicals were purchased from Sigma-Aldrich (Munich, Germany), Merck KGaA (Darmstadt, Germany) or Roth (Karlsruhe, Germany) unless otherwise stated.

### Cell culture

Human HepG2 and mouse Hepa 1.6 hepatocellular carcinoma cell lines were purchased from ATCC (Manassas, USA). Cells were seeded on 6-well plates in 10% (v/v) FBS DMEM containing 4.5 g/l glucose. Next day medium was switched for 0.5% (v/v) FBS DMEM either with glucose at 1.0/4.5 g/l or glucose deprived. Cells were cultured for another 48 h and then collected for RNA extraction or used in *N*-reduction assay.

### Cellular Protein extraction

Cells were washed with ice cold PBS, scratched and collected in a tube. PBS was removed after centrifugation and cells were resuspended in ice cold lysis buffer (1% (v/v) Nonident P-40, 150 mM NaCl, 50 mM Tris, pH 8.0, protease inhibitor mixture (complete ULTRA; Roche Diagnostics, Laval, Canada)) and shaken for 1 h at 4°C. Lysates were centrifuged and supernatant stored at −80°C for later use in Western Blot.

### Animals

Male C57BL/6W mice and B6.V-*Lep^ob^/J* mice (ob/ob), homozygous for leptin gene mutation, were housed under temperature (21±2°C) and humidity (55±10%) controlled conditions, with a 12 h light/dark cycle and ad libitum access to food and water.

### Ethics statement

Mice were housed in the Department of Genetics and Laboratory Animals Breading, at the Maria Sklodowska-Curie Memorial Cancer Center and Institute of Oncology. Experimental protocol was approved by the 2^nd^ Local Ethical Committee for Animal Research in Warsaw, Poland. For benzamidoxime admistration, each animal was given anesthesia by intraperitoneal administration of pentobarbital at a dose of 40 mg/kg. Animals were euthanized by isoflurane overdose followed by cervical dislocation.

### Animal fasting experiment design

All animals were fed regular diet (10% of calories from fat), containing 19.2% protein, 67.3% carbohydrate, and 4.3% fat (D12450B; Research Diets, New Brunswick, USA). In a first experiment 24 animals were used and 12 of them were deprived of food for a period of 18 h before sacrifice followed by liver collection. In a second experiment 28 animals were used and 14 of them were deprived of food for a period of 24 h prior to benzamidoxime administration. Benzamidoxime was reconstituted in water (injection grade). Each animal was given anesthesia by intraperitoneal administration of pentobarbital at a dose of 40 mg/kg. Benzamidoxime was then delivered by intravenous injection at 20 mg/kg dose and 30 minutes later mice were sacrificed followed by immediate collection of liver and blood for plasma. Samples were snap-frozen and stored at −72°C until use.

### Animal HFD experiment design

Both one group of C57BL/6W mice (control) and ob/ob-mice were fed regular diet (10% of calories from fat), containing 19.2% protein, 67.3% carbohydrate, and 4.3% fat (D12450B; Research Diets, New Brunswick, USA). A second group of C57BL/6W mice were fed HFD (60% of calories from fat), containing 26.2% protein, 26.3% carbohydrate, and 34.9% fat (D12492; Research Diets, New Brunswick, USA). At 16 weeks of age, mice were sacrificed followed by immediate collection of livers. Samples were snap-frozen and stored at −72°C until use.

### Preparation of mice liver homogenates

Mice livers were cut into pieces and homogenized using a Potter S homogenizer (Satorius, Goettingen, Germany) in ice cold buffer containing 0.25 M saccharose, 1 mM EDTA, 10 mM potassium dihydrogene phosphate and 1 mM dithiotreitol with pH 7.4. Samples were frozen and stored at −80°C for later use.

### Determination of protein content

For both homogenates and cell culture lysates, protein content was determined using bicinchoninic acid (BCA) protein assay kit (Pierce, Rockford, USA) according to manufacturer’s protocol.

### RNA extraction and expression studies

Total RNA was isolated from liver and cell culture samples using the RNeasy Plus Mini Kit (Qiagen, Hilden, Germany) or TRIzol Plus RNA Purification Kit (Life Technologies, Carlsbad, USA), respectively, followed by on-column DNAse I digestion. One µg of total RNA and random hexamers were used in cDNA synthesis with Superscript III according to manufacturer’s protocol (Life Technologies, Carlsbad, USA). Levels of specific mRNAs were assessed by quantitative real-time PCR (qRT-PCR) using primer pairs at final concentration of 200 nM (Table 1). QRT-PCR was carried out in ABI 7900HT Fast Real-Time PCR System with Sensimix SYBR kit (Bioline, Boston, USA) using standard cycling conditions at 40 cycles consisting of 15 s of denaturation at 95°C and hybridisation for 1 min at 60°C in a 384-well reaction plate. 60S acidic ribosomal protein P0 (RPLP0) mRNA expression was used as reference mRNA for HepG2 samples. Geometric mean of Ct values for Mcoln1(Mucolipin-1) and Hmbs (hydroxymethylbilane synthase) mRNAs was used to normalize gene expression in Hepa 1.6 and liver samples. Mean gene expression was calculated with delta-delta Ct (ddCt) method.

**Table 1:** Primers used for expression studies.

Species	Gene name	Forward (5′→3′)	Reverse (5′→ 3′)
Human	RPLP0	GCAATGTTGCCAGTGTCTG	GCCTTGACCTTTTCAGCAA
Human	mARC1	ACTCCAGTGTCTGGGTCCAC	CAGGCCAAATATTGTGGTGA
Human	mARC2	CAGCACAAAATACTGCCCAA	AACCGCTGGAAACACTGAAG
Human	CYB5B	GCTTGTTCCAGCAGAACCTC	GGAACTGTGGCTTGTGATCC
Human	CYB5R3	AGGATGTGCTGGGGTGAC	AGCCCGGACATCAAGTACC
Human	PCK1	GATTGTGTTCTTCTGGATGGT	TGACGCACAAGGTCATTTAAG
Mouse	Hmbs	AAGGGTTTTCCCGTTTGC	TCCCTGAAGGATGTGCCTA
Mouse	Mcoln1	CCACCACGGACATAGGCATAC	GCTGGGTTACTCTGATGGGTC
Mouse	mARC1	CATTTGCCGAGAACTTCTGGG	ATGCAGCTCAGTGGGTCAG
Mouse	mARC2	GAACCTGTCGCGCACTTTG	GGATCTACCCGATCAAGTCCT
Mouse	CYB5B	AGCTTTCAGTTGCATCAGCAC	GAGCCCTCCGTCACCTACTA
Mouse	CYB5R3	CAGGCCGCAACAGGATATCT	AACGACCACACCGTGTGCTA
Mouse	PCK1	GCCTTCCACGAACTTCCTCAC	CTGCATAACGGTCTGGACTTC

### Western Blot analysis

For SDS-PAGE, separation gels containing 12.5% (v/v) of polyacrylamide were used according to the method of Laemmli [17]. Protein samples were diluted to equal protein concentrations and pretreated with β-mercaptoethanol for 10 min at 100°C prior to loading onto the gel. After separation, proteins were blotted on a PVDF transfer membrane (Amersham Hybond P Membrane; GE Healthcare, Buckinghamshire, UK) and blocked for at least one hour in 5% (m/v) milk powder in TBS buffer containing Tween 20 (TBST). For immuno-blot analysis, the following primary antibodies were used: anti-CYB5R3-antibody (HPA001566; Sigma-Aldrich, St. Louis, USA), anti-mARC2-antibody (HPA015085; Sigma-Aldrich, St. Louis, USA), anti-CYB5B-antibody (HPA007893; Sigma-Aldrich, St. Louis, USA), anti-PCK1-antibody (ab28455; Abcam, Cambridge, UK), anti-MOSC1-antibody (AP9754c; Abgent, San Diego, USA) for of HepG2 lysates or anti-MOSC1-antibody (ABIN503067; antibodies-online GmbH, Aachen, Germany) for murine liver and Hepa 1.6 lysates. Either anti-histone H3-antibody (ab1791; Abcam, Cambridge, UK) or anti-calnexin-antibody (AP03028SU-N; Acris, Herford, Germany) were used as loading control. Incubation with primary antibodies was carried out over night at 4°C; membranes were then washed with TBST. For visualization, a secondary horseradish peroxidase-conjugated anti-rabbit-immunoglobuline-G-antibody (Jackson Immuno Research Laboratories, Suffolk, UK) was used, incubation time was one hour. After washing in TBST, chemiluminescence detection was carried out with Amerscham ECL Plus Western Blotting Detection System (GE Healthcare, Buckinghamshire, UK) according to the manufacturer's instructions.

### *N*-reductive activity assay for mice liver homogenates

*N*-reductive activity was determined by measuring the reduction of the model compound benzamidoxime to benzamidine. 50 ng of homogenate were incubated in a total volume of 150 µl 100 mM potassium phosphate buffer containing 3 mM benzamidoxime at 37°C. After 3 min of preincubation, the reaction was initiated by addition of 1 mM NADH and stopped after 20 min by addition of 150 µl ice-cold methanol. Samples were centrifuged and the supernatant was analyzed via HPLC.

### *N*-reductive activity assay for cell cultures

For *N-*reductive activity assay in cell cultures, cell culture medium was removed and cells were gently washed and preincubated for 15 min with benzamidoxime-free incubation buffer (Hanks balanced salt solution and 10 mM HEPES, pH 7.4) at 37°C. The buffer was carefully replaced by 1 ml of incubation buffer containing 5 mM benzamidoxime and incubation was carried out for 120 min. Afterwards supernatant was frozen at −20°C. Later samples were centrifuged to remove cellular contamination and analyzed via HPLC.

### Determination of benzamidine in mice plasma and homogenates

Plasma was mixed with two aliquots and homogenates were mixed with twenty aliquots of acetonitrile and shaken for 30 min at 4°C. After centrifugation for 30 min, the supernatant was dried using a Christ Alpha 2–4 freeze dryer (Martin Christ Gefriertrocknungsanlagen GmbH, Osterode am Harz, Germany). The resulting lyophilisate was resolved in mobile phase for HPLC analysis (10 mM octylsulfonate sodium salt, 17% acetonitrile), shaken for 45 min at 4°C and cleared again by centrifugation for 30 min. The resulting supernatant was analyzed by HPLC.

### Quantification of the metabolite benzamidine by HPLC

In case of *N*-reductive activity assay with cell cultures, benzamidine was quantified using a Phenomenex Gemini (150×4.6 mm) 5 µM C_18_ column with a Phenomenex C_18_ 4×3 mm guard-column (Phenomenex, Aschaffenburg, Germany). The mobile phase consisted of 50 mM ammonium acetate buffer, pH 7.0 and 10% (v/v) methanol. Flow rate was 1 ml/min at room temperature and detection was carried out at 229 nm. Retention time for benzamidine was 5.2±0.1 min and for benzamidoxime 12.0±0.2 min.

In case of *in vitro N*-reductive activity assay with homogenates and quantification of benzamidine in plasma and homogenates of animals that were treated with benzamidoxime, quantification was carried out as follows: A LiChroCHART 250×4 mm column with LiChrospher 60 RP-select B (5 µm) and a RP-select B 4×4 mm guard column (Merck kGaA, Darmstadt, Germany) was used. The mobile phase consisted of 10 mM sodium octylsulfonate sodium salt and 17% (v/v) acetonitrile. Flow was kept isocratically at 1 ml/min at room temperature and detection was carried out at 229 nm. Retention time for benzamidine was 25.5±0.2 min and for benzamidoxime 6.9±0.1 min.

### Statistical analyses

Differences in pair wise comparisons were evaluated using the *U*-test in GraphPad Prism 5 (GraphPad Software, Inc.; CA, USA). A *p*-value of less than 0.05 was considered significant (*), (**) = *p*-value <0.001(**), (***) = *p*-value <0.0001.

## Results

### Glucose deprivation downregulates mARC complex components abundance and its activity in cell culture

**Figure 2:**
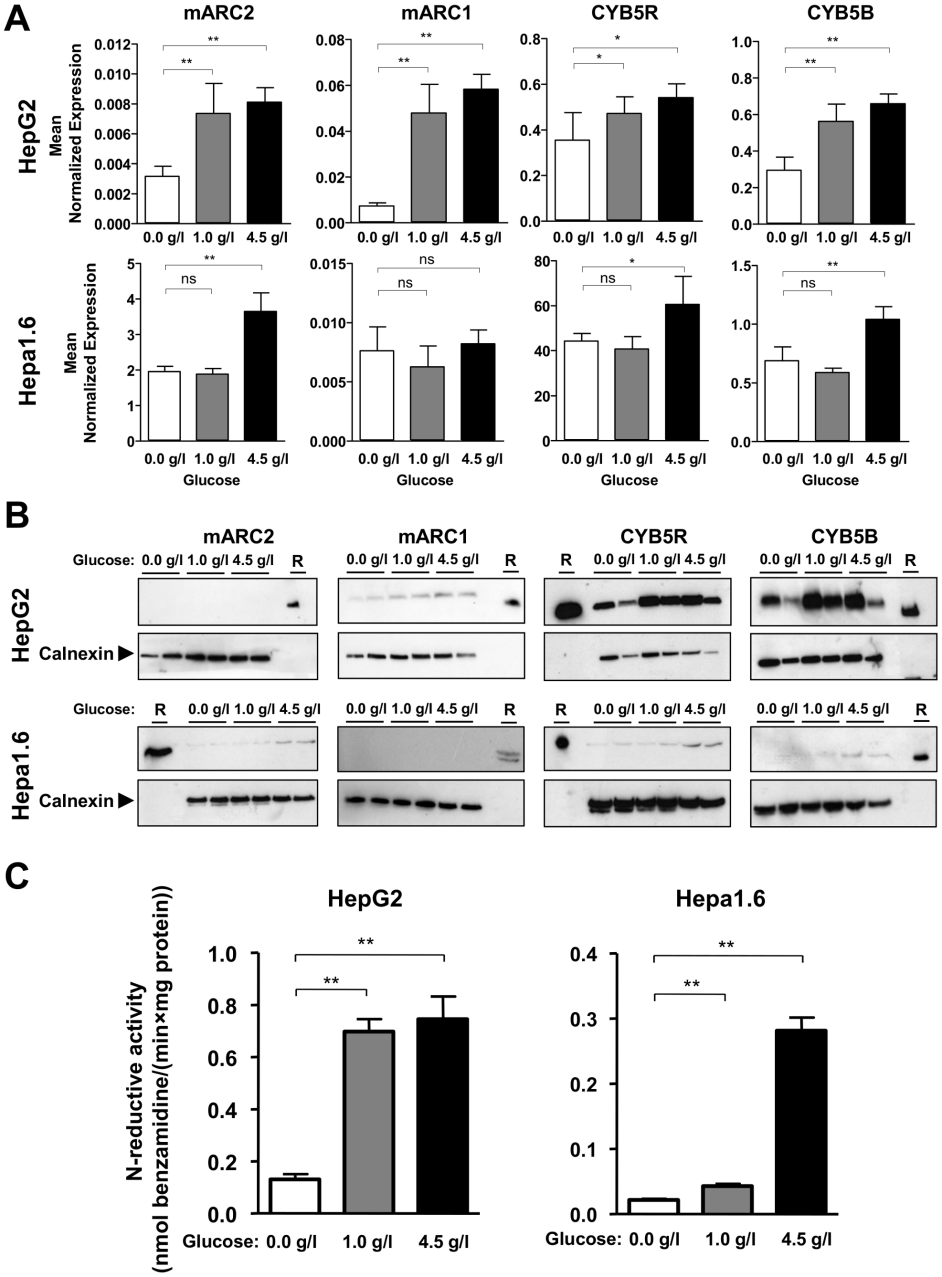
Glucose-dependent changes with expression, abundance and activity of the *N*-reductive system in HepG2 and Hepa 1.6 cell lines. **A** Expressions of mARC2, mARC1, CYB5R and CYB5B in HepG2 and Hepa 1.6 cell lines cultured without or with 1.0/4.5 g/l glucose determined by qRT-PCR. Statistical significance was assessed by the U-test. *p*-values <0.05 were considered significant (*); (**) *p*-value <0.001, ns = not significant. **B** Protein levels of mARC2, mARC1, CYB5R and CYB5B in hepatoma cell lines cultured without or with 1.0/4.5 g/l glucose, examined by Western Blot. R = recombinant proteins/control **C** *N*-reductive activity determined in in hepatoma cell lines by determining the reduction of model compound benzamidoxime. The resulting metabolite benzamidine was quantified by HPLC analysis. Determined activities are means ± SD of six biological samples, each measured as duplicates. Statistical significance was assessed by the U-test. *p*-values <0.05 were considered significant (*); (**) *p*-value <0.001.

To test if the mARC-containing enzyme system is influenced by the state of nutrient depletion, we used *in vitro* human HepG2 and mouse Hepa 1.6 hepatocellular carcinoma cell line models cultured with and without glucose. Glucose withdrawal is regarded as a condition that could mimic physiological fasting in cell culture [18]. Hepatocellular carcinoma cell lines were chosen as the liver is the main organ of energy metabolism. Cells were cultured in cell culture medium without (0.0 g/l) or with either 1.0 g/l or 4.5 g/l glucose and gene and protein expression measurements were performed. Gene expressions for mARC1, mARC2, CYB5R and CYB5B were clearly enhanced in HepG2 cells that were grown either in 1.0 g/l or 4.5 g/l glucose. For Hepa 1.6 cells only 4.5 g/l glucose substantially increased mARC2, CYB5R and CYB5B mRNA abundance without effect on mARC1 transcription level (Figure 2A). Transcripts changes for HepG2 cell line were mirrored in protein abundances where mARC1, CYB5R and CYB5B protein levels measured with Western Blot were higher in cells cultured with glucose. mARC2 was not detectable in any cell lysates. The protein levels examined in Hepa 1.6 cells corresponded with mRNA changes observed for culture conditions with 4.5 g/l glucose. In these cells mARC1 protein could not be detected (Figure 2B). Both hepatoma cell lines were next incubated with model compound benzamidoxime and the reduced metabolite benzamidine was determined in cell culture supernatant to determine *N*-reductive activity. Cell lines grown in medium containing 1.0 g/l or 4.5 g/l glucose showed significantly higher *N*-reductive activity compared to cells grown without glucose (Figure 2C).

### Fasting in mice down-regulates mARC complex components abundance and its activity

**Figure 3:**
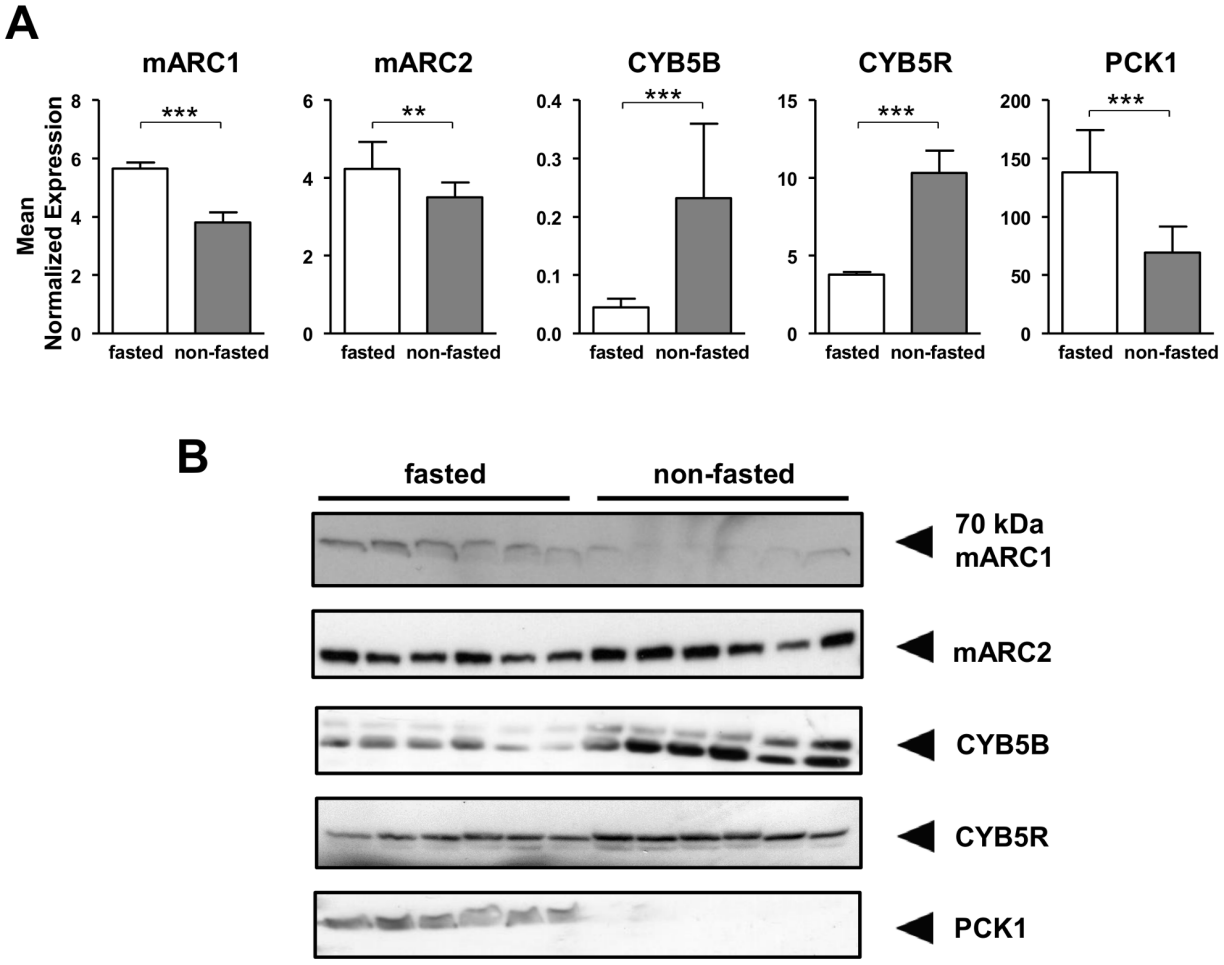
Effects of fasting on expression and protein abundance of the *N*-reductive system in mice. Two groups of 12 C57BL/6W mice were fed with regular diet, one group were food deprived for 18 h (fasted) before sacrifice and liver collection, the second had full access to food and water (non-fasted). **A** Expressions of mARC1, mARC2, CYB5B, CYB5R and PCK1, determined with qRT-PCR, normalized on geometric mean of Ct values for Mcoln1 (Mucolipin-1) and Hmbs (hydroxymethylbilane synthase). Statistical significance was assessed by the U-test. *p*-values <0.05 were considered significant; (**) *p*-value <0.001; (***) *p*-value <0.0001. **B** Protein levels of mARC1, mARC2, CYB5B, CYB5R and PCK1 examined by Western Blot. Each sample consisted of equal protein amount of two individuals.

**Figure 4:**
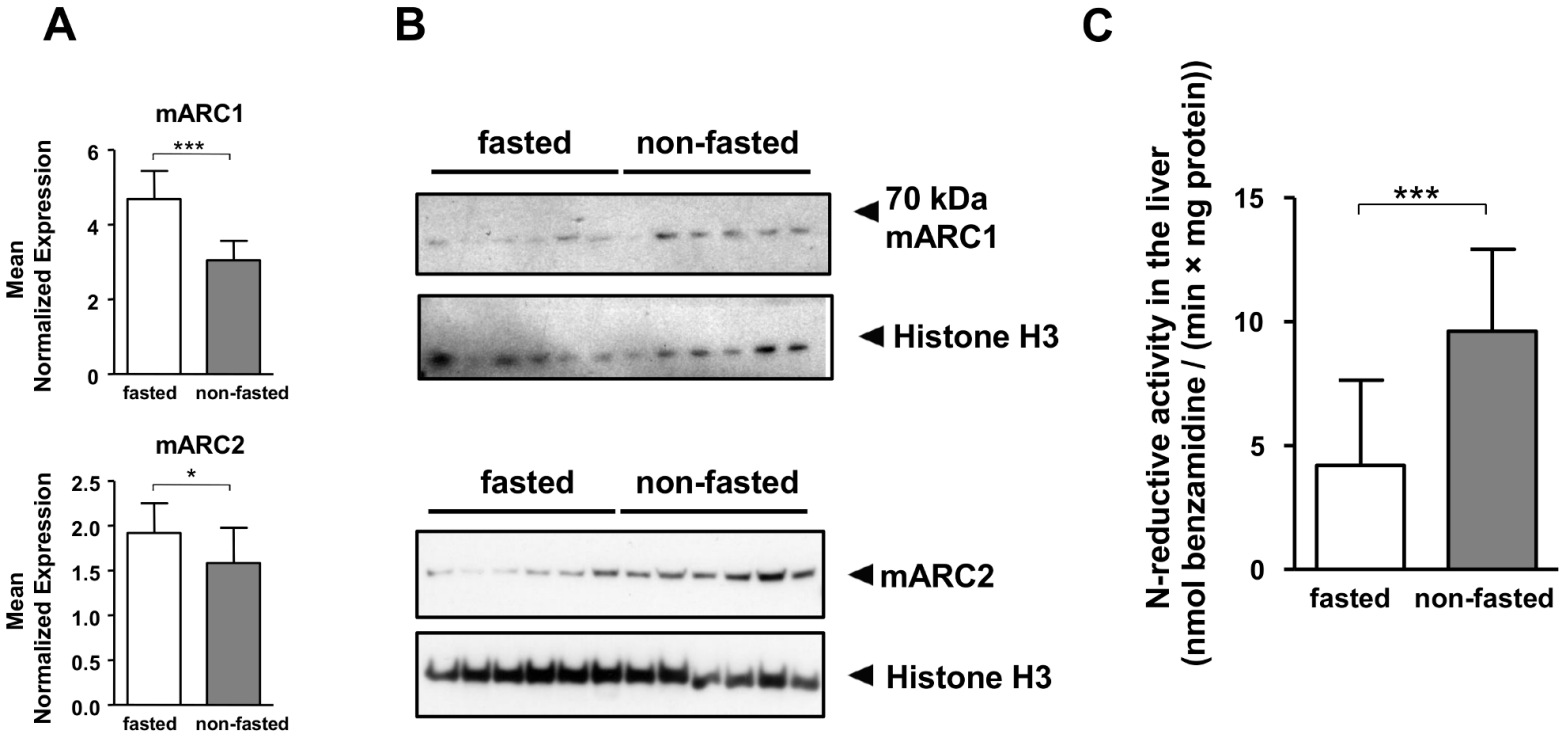
24 h fasting decreases mARC protein levels and *N*-reductive activity in mice. Two groups of 14 C57BL/6W mice were fed with regular diet, one group were food deprived for 24 h (fasted) before sacrifice and liver collection, the second had full access to food and water (non-fasted). **A** Expressions of mARC1 and mARC2, determined with qRT-PCR, normalized on expression of Mcoln1 (Mucolipin-1) and Hmbs (hydroxymethylbilane synthase). Statistical significance was assessed by the U-test. *p*-values <0.05 were considered significant (*); (**) *p*-value <0.001; (***) *p*-value <0.0001. **B** Protein levels of mARC1, mARC2 and histone H3 (loading control) examined by Western Blot. Each sample consisted of equal protein amount of two individuals. **C** *N*-reductive activity determined by the reduction of model compound benzamidoxime in liver homogenate. The resulting metabolite benzamidine was quantified by HPLC analysis. Determined activities are means ± SD of 14 biological samples, each measured as duplicates. Statistical significance was assessed by the U-test. *p*-values <0.05 were considered significant (*); (***) *p*-value <0.0001.

To study the influence of food deprivation on the mARC protein and its electron transfer partner proteins *in vivo*, mice experiments with 18 h and 24 h food deprivation were carried out. Livers were examined, as the liver is a center of metabolic conversions and energy metabolism and changes due to food deprivation are likely to occur in this organ. Gene expression and protein abundances for CYB5B and CYB5R were clearly decreased in the fasting group after both 18 and 24 h of food deprivation. Marker protein PCK1 was enhanced in the fasting groups of both experiments and thus confirmed the fasting state (Figure 3 & Figures S1A, S2). Gene expression for both mARC1 and mARC2 was enhanced in the fasted groups (Figures 3A & 4A); however no changes in protein levels for mARC2 were detectable after 18 h of fasting (Figure 3B & Figure S1A). In contrast, after 24 h of fasting, decreased abundance of mARC2 was detectable in the fasted group compared to control (Figure 4B). For mARC1, an antibody with reactivity against mARC1 detected a protein with increased abundance upon 18 h of fasting but with decreased protein levels with 24 h of fasting. However, the molecular weight of the detected protein was about 65 kDa and thus not the predicted molecular weight of 37 kDa of mARC1 (Figures 3B & 4B, Figure S1A). The *N*-reductive activity was determined in an *in vitro* assay with liver homogenates, where the reduction of the model compound benzamidoxime to benzamidine is analyzed. The *N*-reductive activity was clearly reduced in mice that underwent 18 h or 24 h of fasting compared to control (Figure 4C & Figure S1B).

### Fasting in mice does not influence concentrations of the metabolite of the N-reduction

**Figure 5:**
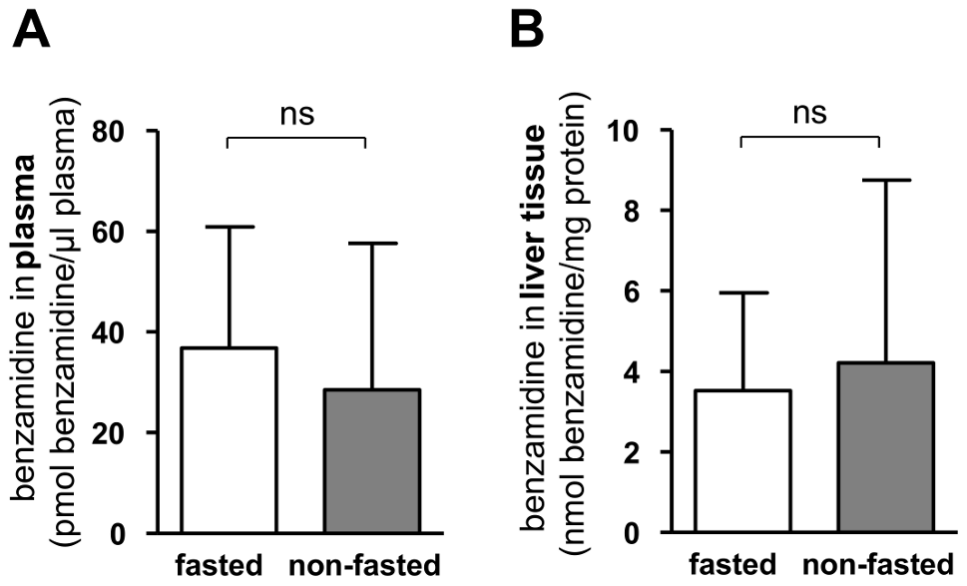
Metabolite concentrations of the *N*-reductive reaction are not affected by fasting in mice. Two groups of 14 C57BL/6W mice were fed with regular diet, one group were food deprived for 24 h (fasted); the second had full access to food and water (non-fasted). After anesthesia, 20 mg/kg benzamidoxime was injected. 30 min later animals were sacrificed followed by plasma liver collection **A** Metabolite concentrations in plasma 30 min after benzamidoxime injection, determined by HPLC analyses after sample work-up. **B** Metabolite concentrations in liver homogenates 30 min after benzamidoxime injection, determined by HPLC analyses after sample work-up. Determined activities are means ± SD of 12 biological samples, each measured as duplicates. Statistical significance was assessed by the U-test. *p*-values <0.05 were considered significant, ns = not significant.

To study whether fasting influences the concentration of the metabolite of the *N*-reductive reaction (benzamidine), benzamidoxime was administered to mice that fasted for 24 h or had full access to food (control). First, we performed a pilot study to set a time of sampling after benzamidoxime administration that indicated rapid metabolite decline in a time dependent manner in both plasma and liver tissue, where mother compound was undetectable in plasma after 1 hour (Figure S3). Based on this data we collected liver and plasma samples 30 minutes after benzamidoxime dosing and then level of benzamidine were determined with HPLC. However, no statistically significant differences for the metabolite benzamidine could be found in plasma or in liver homogenates of fasted and control animals (Figure 5).

### HFD but not hyperphagia influences mARC abundance and its activity in mice

**Figure 6:**
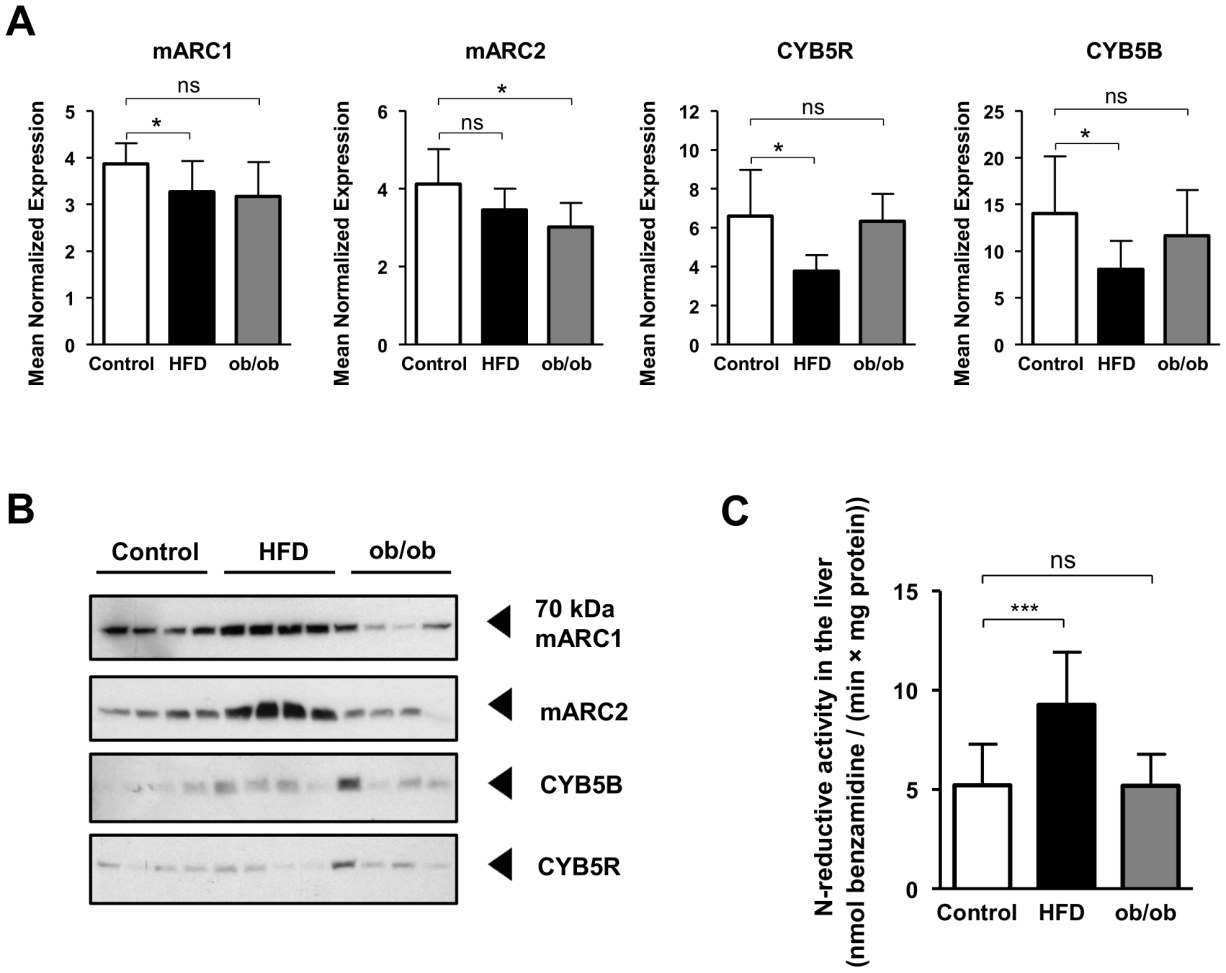
HFD but not hyperphagia increases mARC abundance and *N*-reductive activity in mice. Both a group of C57BL/6W and ob/ob-mice were fed with regular diet, another group of C57BL/6W mice was fed with HFD. Mice were sacrificed and livers collected. **A** Expressions of mARC1, mARC2, CYB5R and CYB5B determined with qRT-PCR, normalized on expression of Mcoln1 (Mucolipin-1) and Hmbs (hydroxymethylbilane synthase). Expression shown as means ± SD of 6 biological samples. Statistical significance was assessed by the U-test. p-values <0.05 were considered significant (*). **B** Protein levels of mARC1, mARC2, CYB5B and CYB5R in liver homogenates examined by Western Blot. **C** *N*-reductive activity determined by the reduction of model compound benzamidoxime in liver homogenate. The resulting metabolite benzamidine was quantified by HPLC analysis. Determined activities are means ± SD of four biological samples, each measured as duplicates. Statistical significance was assessed by the U-test. p-values <0.05 were considered significant (*); (***) p-value <0.0001; ns = not significant.

To test the influence of HFD and hyperphagic behavior on the *N*-reductive system we used liver tissue samples of HFD-fed and ob/ob-mice described in recent studies by Nesteruk *et al.* and Hennig *et al.* [19], [20]. Livers of HFD-fed mice showed decreased mRNA expressions for mARC1, CYB5B and CYB5R, but no changes for mARC2 compared to control (Figure 6A). Contrary, an increased protein abundance for mARC2 was detected, whereas no differences in the abundance of CYB5B and CYB5R could be demonstrated. An antibody with the reactivity against mARC1 detected a protein with increased abundance due to HFD. However the molecular weight of about 65 kDa of the detected protein did not fit the predicted molecular weight of mARC1 with 37 kDa (Figure 6B & Figure S4). Thus, as with mice experiments with fasting, mARC mRNA and protein level do not show same tendencies. The *N*-reductive activity, determined with the reduction of the model compound benzamidoxime to benzamidine, was also enhanced in livers of HFD-fed mice (Figure 6C). In contrast to the HFD-fed mice, no changes with respect to protein abundance or *N*-reductive activity compared to control were detectable for obese hyperphagic ob/ob-mice (Figure 6 B–C & Figure S4).

## Discussion and Conclusion

### Glucose deprivation downregulates mARC complex components abundance and its activity in human and murine hepatoma cell lines

Expressions, protein abundance for all three proteins of the *N*-reductive system and *N*-reductive activity increase both in mouse Hepa 1.6 and human HepG2 cell culture fasting models in a glucose-dependent manner, except mARC1 mRNA in Hepa 1.6. However, in mouse Hepa 1.6 mARC1 and in human HepG2 mARC2 were not detectable on protein levels (Figure 2). In both cases, proteins were probably below detection limit. In Hepa 1.6 determined trancript level of mARC1 was about 400 times lower than for mARC2. For HepG2, mARC2 mRNA was about 7 times lower relativly to mARC1 (Figure 2A) and BioGPS database [21] describes as well only faint expression of mARC2 in HepG2 cell line compared to mARC1 (BioGPS gene numbers 64757 and 54996).

Yet, we can demonstrate that all three components of the *N*-reductive system are regulated in a glucose-dependent manner and support the hypothesis that the enzyme system is linked with energy metabolism. Our data is in consistence with studies showing mARC2 to be regulated glucose-dependent in kidney cell culture [13].

### Fasting in mice down-regulates mARC complex components abundance and its activity

Expression for CYB5B and CYB5R was clearly reduced in both experiments under fasting conditions, mirrored by the proteins’ abundances (Figure 3 & Figures S1–S2). Expression for mARC2 showed opposite tendencies and increased with fasting (Figure 3A & Figure 4A). Specifically, with 18 h of fasting no changes were detected while 24 h fasting resulted in decreased protein levels for mARC2 (Figures 3B & 4B, Figure S1). With mARC1, mRNA expression increased with 18 h fasting was mirrored by increased protein levels of a protein assumed to be mARC1. In contrast 24 h of fasting resulted in decreased protein levels although mRNA levels were significantly enhanced. *N*-reductive activity was clearly reduced by fasting in both experiments (Figure 4C & Figure S1B).

With respect to detection of mARC1 protein levels, a protein was detected at about 65 kDa, which does not fit the predicted molecular weight of 37 kDa for mARC1. Wahl *et al.* already demonstrated the same phenomenon and detected a protein with a mARC1-specific antibody at this molecular weight in murine tissues [3]. Moreover, the detected protein follows the same pattern as mARC2 upon 24 h of fasting and HFD in mice. Thus we assume the protein to be mARC1 with an increased molecular weight as a result of unknown posttranslational modifications as already hypothesized by Wahl *et al.* [3]. However, it is also possible that the detected signal is not related to mARC1 but stems from an unspecific reaction of the antibody.

The protein levels of the protein assumed to be mARC1 are increased with 18 h of fasting but decreased after 24 h of fasting, indicating a fast breakdown of the protein with prolonged fasting time. Thus protein turnover is different for the three components of the *N*-reductive system. This phenomenon is as well described for another three component system composed of cytochrome b5 and its reductase together with stearyl-CoA desaturase. The latter undergoes more rapid turnover than the other two members of the system for regulatory reasons [22]. The same mechanism could also be assumable for mARC-containing enzyme system. Reasons or mechanisms for the initial increase of the assumed mARC1 protein due to fasting followed by its decrease with prolonged fasting time are not yet understood, as well as the interplay of mARC1 and mARC2 during fasting.

Surprisingly, the abundances of mARC2 and mARC1 mRNA and protein levels in mice show opposite tendencies due to 24 h of fasting (Figure 4A–B). This may indicate a posttranscriptional or posttranslational regulation that is so far unknown. Discrepancies and even opposite expression patterns for mRNA and protein levels have already been described for other proteins [23]–[25]. These findings indicate that further studies on mARCs must not solely be based on RNA data but also on protein levels to be reliable. Nevertheless, mARC-proteins abundance and complex activity levels after 24 h of fasting match regarding tendencies. With respect to the 18 h fasting period we assume that both reduced levels of CYB5B and CYB5R are responsible for decreased *N*-reductive activity, regardless of the stable levels for mARC2 and increased levels for the assumed mARC1. CYB5B has been proven to be essential for *N*-reductive activity *in vitro* enzyme assay and cell culture [4], [5], as well as CYB5R *in vitro*, but only faint amounts of this protein are necessary [5], [6]. The latter might be the reason why others could not prove an involvement of CYB5R in *N*-reduction in cell culture [4]. In case of the 24 h fasting period, all three components of the enzyme system show decreased abundance in fasted mice compared to control and are thus likely to cause a decline in *N*-reductive activity.

Concerning the two isoforms of the mARC protein, mARC1 and mARC2, our group [5] already hypothesized that the isoform mainly involved in the *N*-reductive activity varies from species to species. For pig and rat, mARC2, but not mARC1 was found in the outer mitochondrial membrane (OMM), the site where the mARC protein is mainly localized [1], [4]. In contrast, human OMM was shown to contain mARC1 [26]. In consistence with this, we demonstrated that changes in mARC2 in murine cell line Hepa 1.6 and changes in mARC1 in human cell line HepG2 result in altered *N*-reductive activity, whereas the corresponding second isoform was below detection limit in both cell lines. Nevertheless, in murine liver tissues, both isoforms seem to be involved in altered *N*-reductive activities.

### Fasting in mice does not influence concentrations of the *N*-reduction metabolite

No differences in metabolite concentrations (benzamidine) in liver homogenates and plasma after benzamidoxime administration could be found between fasted and non-fasted mice (control) (Figure 5). Thus, though all three components of the *N*-reductive system are decreased due to fasting, the metabolic conversion is still fast enough and probably complete to results in equal metabolite levels for both states. With respect to testing of new drug candidates, protocols for animal testing in rodents may differ regarding feeding and fasting procedures. However, based on this data, the results should still be comparable regarding the metabolite concentrations of substances that are reduced by the *N*-reductive system.

With respect to prodrug activation, changes in activity of the proteins due to nutrition state would have been crucial, as the enzyme system is applied in the activation of amidoxime prodrugs [10]. Yet, based on this experiment, we expect no influence of fasting on the *in vivo* activation of mARC substrates with a metabolic conversion comparable to benzamidoxime. Nevertheless, prodrug candidates with lower or even faint conversion rates should be tested regarding influence of fasting on tissue and plasma metabolite levels.

### HFD but not hyperphagia influences mARC abundance and activity in mice

Mice fed with HFD showed increased abundance for mARC2 and a protein assumed to be mARC1 and as a consequence increased *N*-reductive activity, whereas ob/ob-mice did not show any changes of the *N*-reductive complex (Figure 6B–C & Fig. S4). With respect to mARC1 protein levels, the detected molecular weight did not fit the predicted molecular weight of the target protein as already discussed with fasting experiments. However, the findings are in consistence with Nesteruk *et al.*, who demonstrated increased mARC1 protein levels in HFD fed mice compared to control with quantitative mass spectrometry technique [19]. This indicates besides the points already discussed with fasting experiments that the protein detected by Western Blot analysis at 65 kDa is supposably a form of mARC1. Again and as already discussed with fasting experiments transcripts and protein levels did not match and showed opposite tendencies for mARC1.

With these findings, we thus demonstrate that changes with mARC2 and probably mARC1 are diet-related and not due to the fact, that the animals develop an obese habitus. Livers of control and HFD fed for 16 weeks old mice did not show any signs of steatosis, whereas ob/ob mice developed mild form [20]. Furthermore, serum glucose was enhanced in HFD mice but not in control and ob/ob-mice [20], suggesting a relation between serum glucose and mARC abundance. Data from other experiments with older mice (48 weeks of age) fed with HFD for 42 weeks could only demonstrate minor differences in abundance and activity of the *N*-reductive enzyme system (Figure S5). HFD fed mice generally gained weight and became obese until about 14^th^ week upon HFD. Beyond that point until 42^nd^ week on HFD mice underwent only minor changes in body weight (Figure S5B). We assume therefore that the *N*-reductive system is influenced by increased blood glucose, an active fat metabolism and fat turn-over as it occurs with growing and weight gaining mice at early stage of HFD administration.

### The mARC proteins and its partners CYB5B and CYB5R are related to lipid metabolism

With this study, we demonstrate for the first time, that the *N*-reductive complex composed of mARCs, CYB5B and CYB5R is regulated by diet. The *N*-reductive system undergoes changes with increased glucose amount in cell culture and due to diet in mice. The fact that the mARC proteins and its electron transfer partners decrease with fasting and that mARC2 and the protein assumed to be mARC1 increases with HFD but not with obese ob/ob-mice, reveals that the proteins are connected with food intake and energy supply. Other recent studies support this conclusion: A single nucleotide polymorphism (SNP) with the mARC1 locus was shown to associate with changes in plasma concentration of both total cholesterol and low density lipoprotein cholesterol [27] and others found an influence of this SNP on both baseline low density lipoproteins and the response to fenofibrate [28]. Neve and coworkers [4] proved an involvement of the mARC2 protein in lipid synthesis in 3T3-adipocytes. Moreover, the mARC2^−/−^ knock out mouse model exhibits decreased total body fat amount [29].

Taken together, it is evident that the function of the mARC proteins is related to lipid metabolism. However, the endogenous substrates and detailed regulation mechanisms of the mARC proteins are still not known and require further research. The enzyme system was proven to be changed with physiological disorder like cancer [11] and diabetes [13]. Thus more detailed knowledge of the physiological function and mechanisms may provide not only basic information about the mARC proteins but also ideas in the further research of these diseases.

## Supporting Information

Figure S1 **Effects of fasting on protein abundance of the** ***N*** **-reductive system in mice.** Two groups of 12 C57BL/6W mice were fed with regular diet, one group were food deprived for 18 h (fasted) before sacrifice and liver collection, the second had full access to food and water (non-fasted). **A** Protein levels of mARC1, mARC2, CYB5B, CYB5R and PCK1 examined by Western Blot, histone-H3 was applied as loading control. Each sample consisted of equal protein amount of two individuals. **B** *N*-reductive activity determined by the reduction of model compound benzamidoxime in liver homogenate. The resulting metabolite benzamidine was quantified by HPLC analysis. Determined activities are means ± SD of 12 biological samples, each measured as duplicates. Statistical significance was assessed by the U-test. *p*-values <0.05 were considered significant (*).
(PDF)

Figure S2 **Effects of fasting on protein abundance of the** ***N*** **-reductive system in mice.** Two groups of 14 C57BL/6W mice were fed with regular diet, one group were food deprived for 24 h (fasted) before sacrifice and liver collection; the second had full access to food and water (non-fasted). Protein levels of CYB5B, CYB5R and PCK1 examined by Western Blot, histone-H3 was applied as loading control. Each sample consisted of equal protein amount of two individuals.
(PDF)

Figure S3 **Depletion of metabolite and mother compound after benzamidoxime administration in mice. A** Metabolite concentrations in plasma and liver homogenate after benzamidoxime administration. Metabolite concentrations were determined by HPLC. Concentrations are means ± SD of two biological samples, each measured as duplicates. **B** Representative HPLC-chromatogram of liver homogenate samples taken 30 min after benzamidoxime administration. **C** Representative HPLC-chromatogramm of liver homogenate samples taken 60 min after benzamidoxime administration.
(PDF)

Figure S4 **HFD but not hyperphagia increases mARC2 and mARC1 abundance in mice.** Both a group of C57BL/6W and ob/ob-mice were fed with regular diet, another group of C57BL/6W mice was fed with HFD. Mice were sacrificed and livers collected. Protein levels of mARC1, mARC2, CYB5B and CYB5R in liver homogenates examined by Western Blot, histone H3 was used as loading control.
(PDF)

Figure S5 **HFD has only minor effects on** ***N*** **-reductive complex abundance and activity in older mice.** One group of C57BL/6W mice was fed with regular, another with HFD chow; mice were sacrificed at 48 weeks of age and livers collected. **A** Protein levels of mARC2, CYB5B and CYB5R in liver homogenates examined by Western Blot, histone H3 was used as loading control. **B** Development of bodyweight during aging of mice. **C** *N*-reductive activity determined by the reduction of model compound benzamidoxime in liver homogenate. The resulting metabolite benzamidine was quantified by HPLC analysis. Determined activities are means ± SD of 5–7 biological samples, each measured as duplicates. Statistical significance was assessed by the U-test. *p*-values <0.05 were considered significant (**) = *p*<0,001.
(PDF)
