# Supplementary figures and images for: The N-Reductive System Composed of Mitochondrial Amidoxime Reducing Component (mARC), Cytochrome b5 (CYB5B) and Cytochrome b5 Reductase (CYB5R) Is Regulated by Fasting and High Fat Diet in Mice
Source: PLoS One. 2014 Aug 21;9(8):e105371. doi: 10.1371/journal.pone.0105371 (PMC4140751; doi:10.1371/journal.pone.0105371)

**A**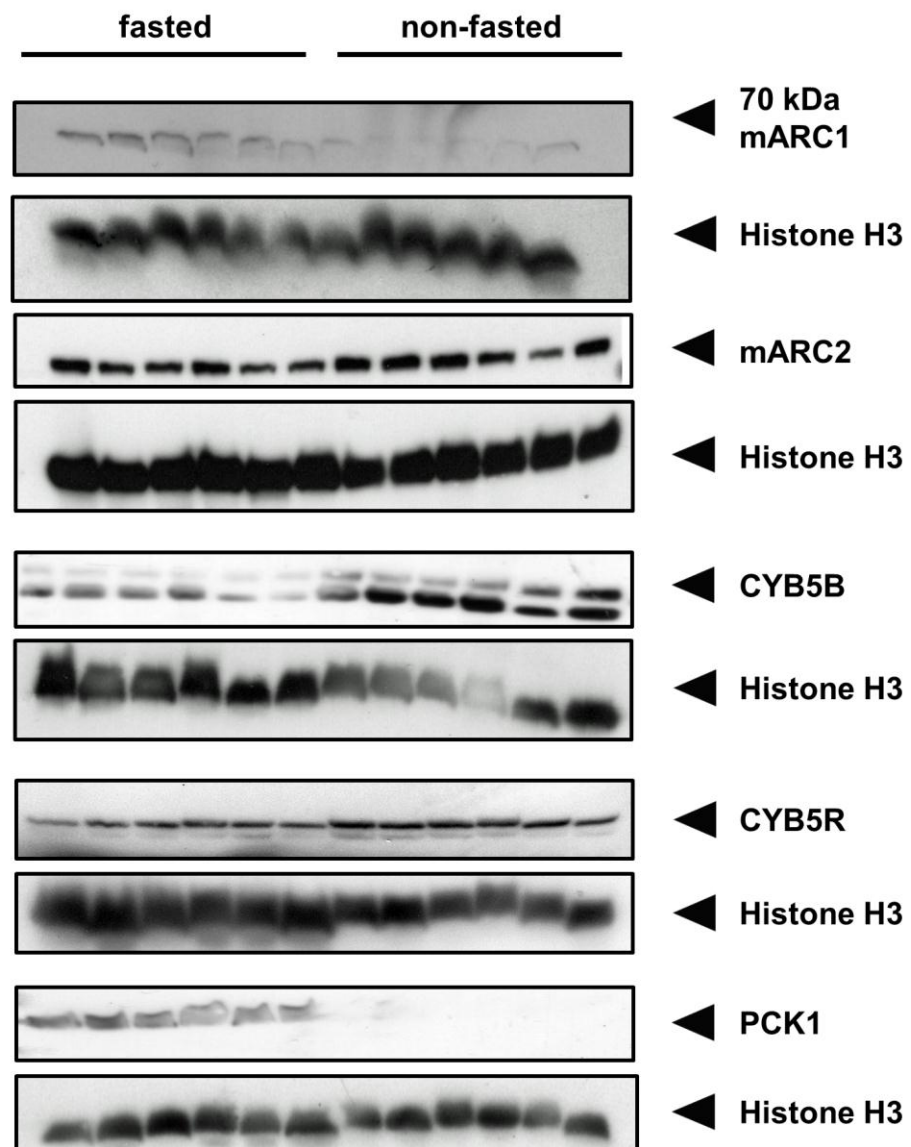**B**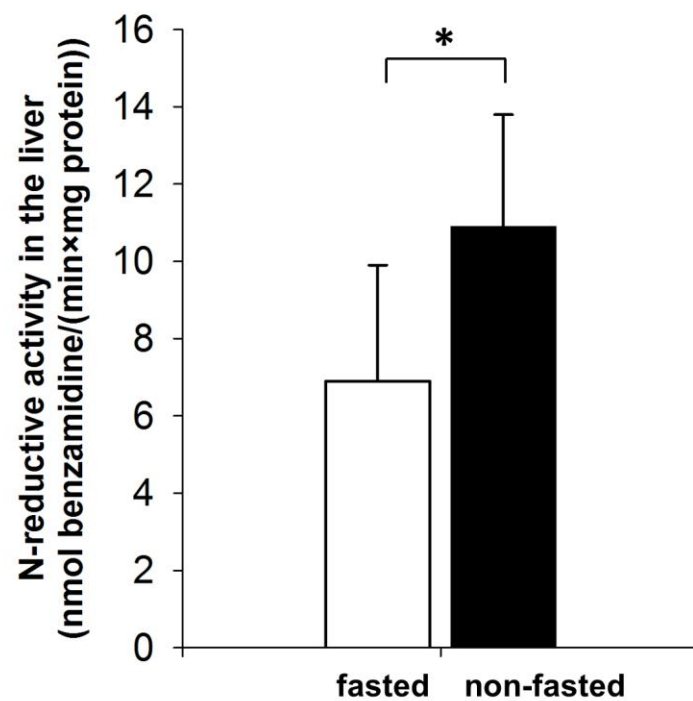

Supplement: Figure S1 — Effects of fasting on protein abundance of the N -reductive system in mice. Two groups of 12 C57BL/6W mice were fed with regular diet, one group were food deprived for 18 h (fasted) before sacrifice and liver collection, the second had full access to food and water (non-fasted). A Protein levels of mARC1, mARC2, CYB5B, CYB5R and PCK1 examined by Western Blot, histone-H3 was applied as loading control. Each sample consisted of equal protein amount of two individuals. B N-reductive activity determined by the reduction of model compound benzamidoxime in liver homogenate. The resulting metabolite benzamidine was quantified by HPLC analysis. Determined activities are means ± SD of 12 biological samples, each measured as duplicates. Statistical significance was assessed by the U-test. p-values <0.05 were considered significant (*). (PDF) [file pone.0105371.s001.pdf]

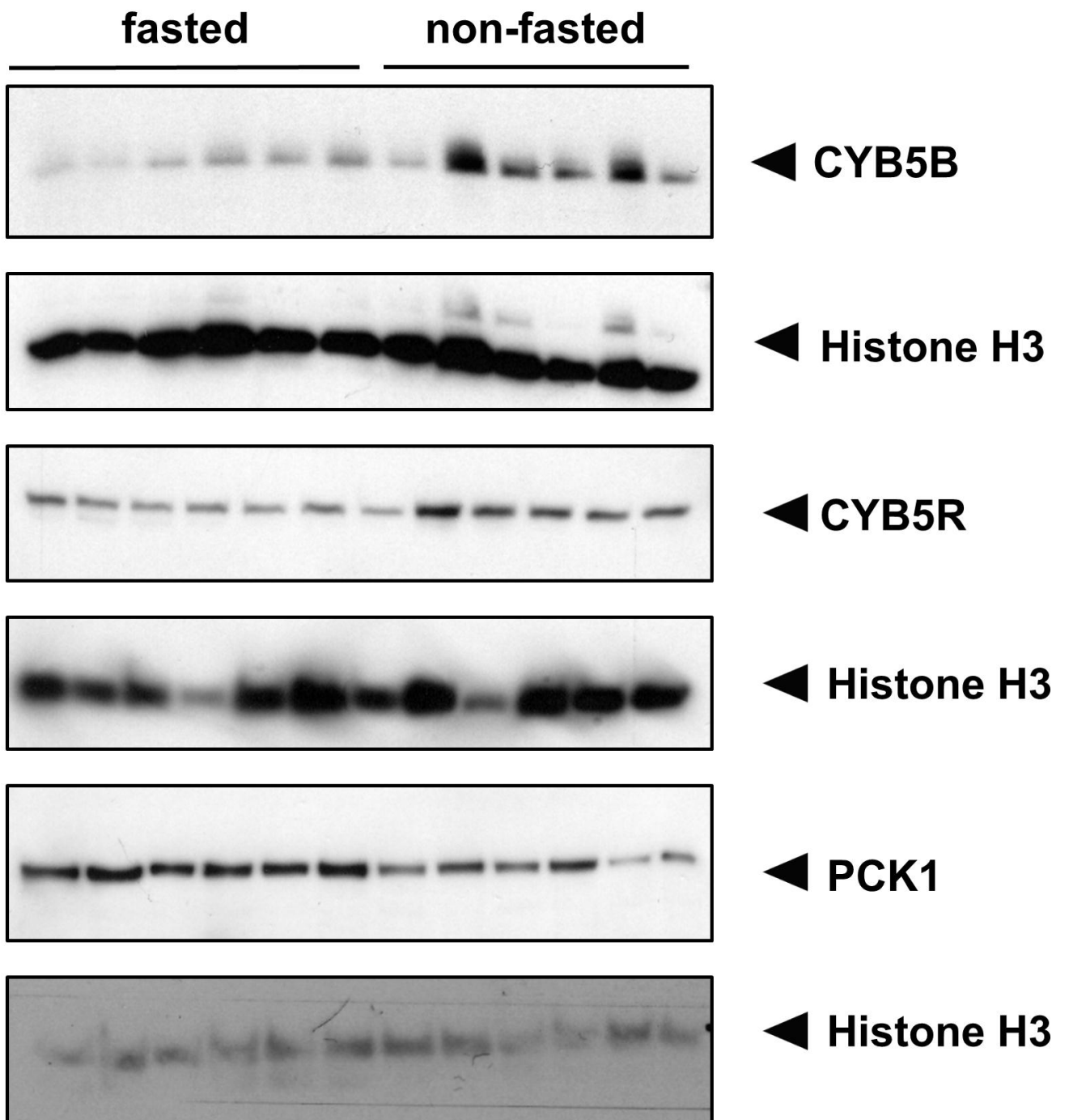

Supplement: Figure S2 — Effects of fasting on protein abundance of the N -reductive system in mice. Two groups of 14 C57BL/6W mice were fed with regular diet, one group were food deprived for 24 h (fasted) before sacrifice and liver collection; the second had full access to food and water (non-fasted). Protein levels of CYB5B, CYB5R and PCK1 examined by Western Blot, histone-H3 was applied as loading control. Each sample consisted of equal protein amount of two individuals. (PDF) [file pone.0105371.s002.pdf]

**A**

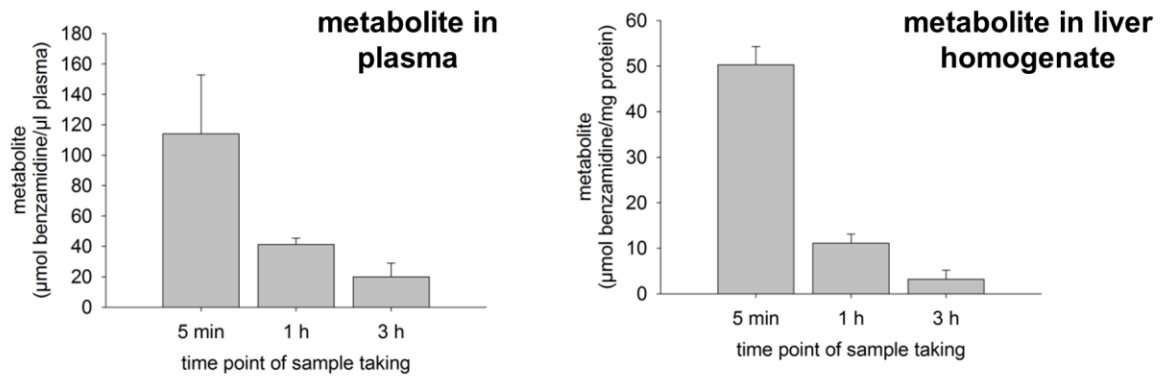

**B**

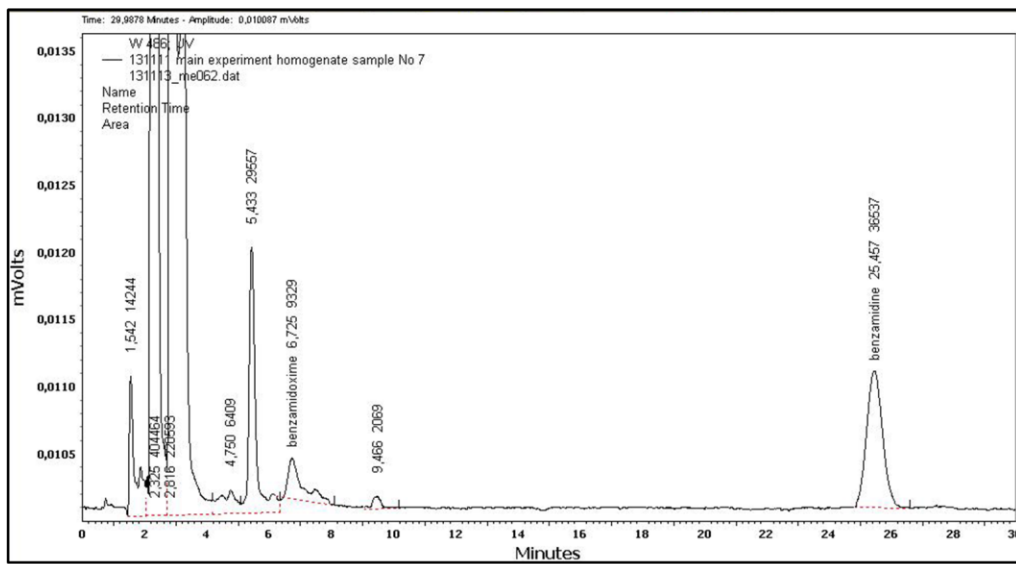

**C**

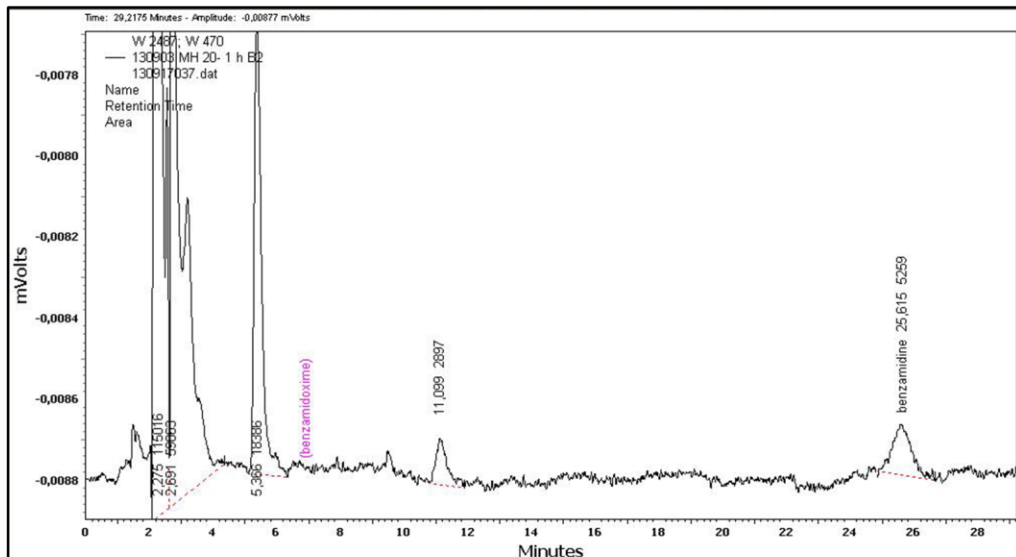

Supplement: Figure S3 — Depletion of metabolite and mother compound after benzamidoxime administration in mice. A Metabolite concentrations in plasma and liver homogenate after benzamidoxime administration. Metabolite concentrations were determined by HPLC. Concentrations are means ± SD of two biological samples, each measured as duplicates. B Representative HPLC-chromatogram of liver homogenate samples taken 30 min after benzamidoxime administration. C Representative HPLC-chromatogramm of liver homogenate samples taken 60 min after benzamidoxime administration. (PDF) [file pone.0105371.s003.pdf]

**Control**

**HFD**

**ob/ob**

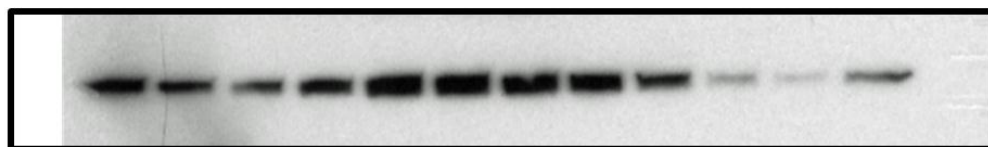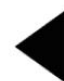

**70 kDa  
mARC1**

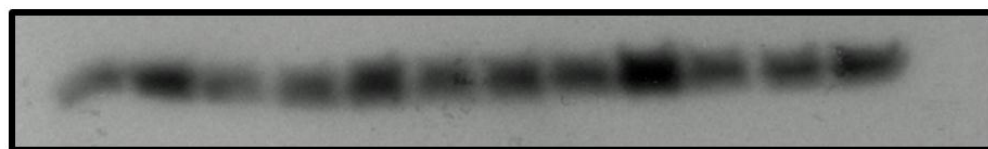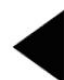

**Histone H3**

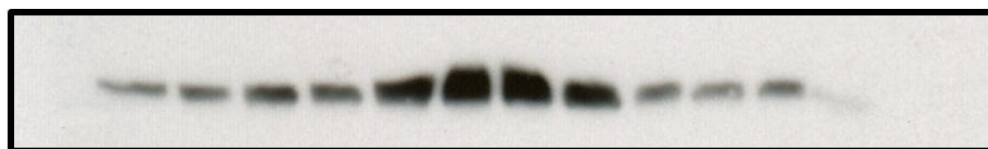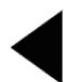

**mARC2**

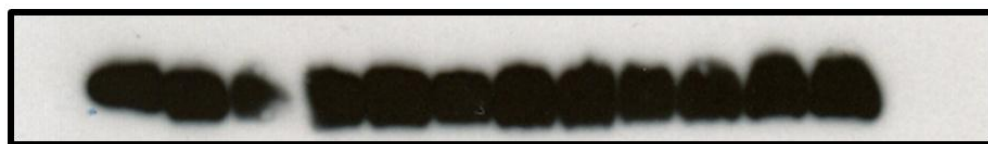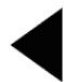

**Histone H3**

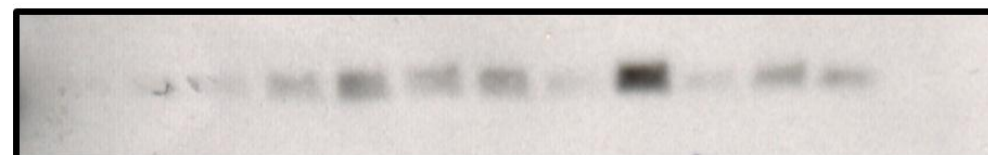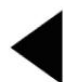

**CYB5B**

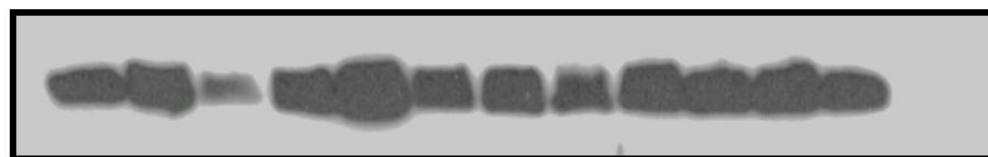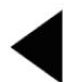

**Histone H3**

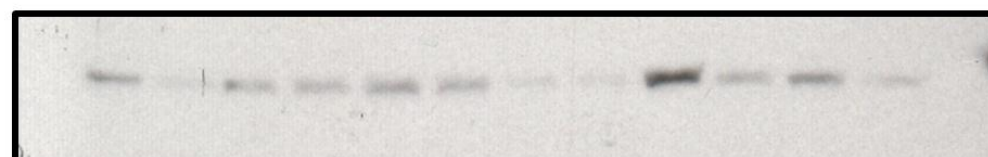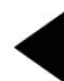

**CYB5R**

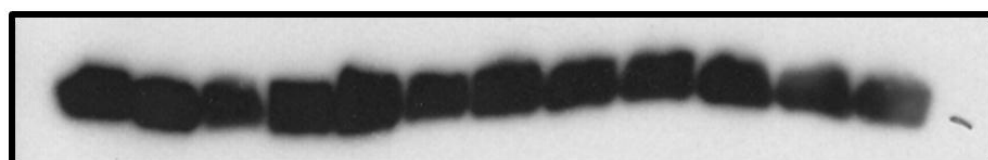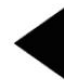

**Histone H3**

Supplement: Figure S4 — HFD but not hyperphagia increases mARC2 and mARC1 abundance in mice. Both a group of C57BL/6W and ob/ob-mice were fed with regular diet, another group of C57BL/6W mice was fed with HFD. Mice were sacrificed and livers collected. Protein levels of mARC1, mARC2, CYB5B and CYB5R in liver homogenates examined by Western Blot, histone H3 was used as loading control. (PDF) [file pone.0105371.s004.pdf]

**A**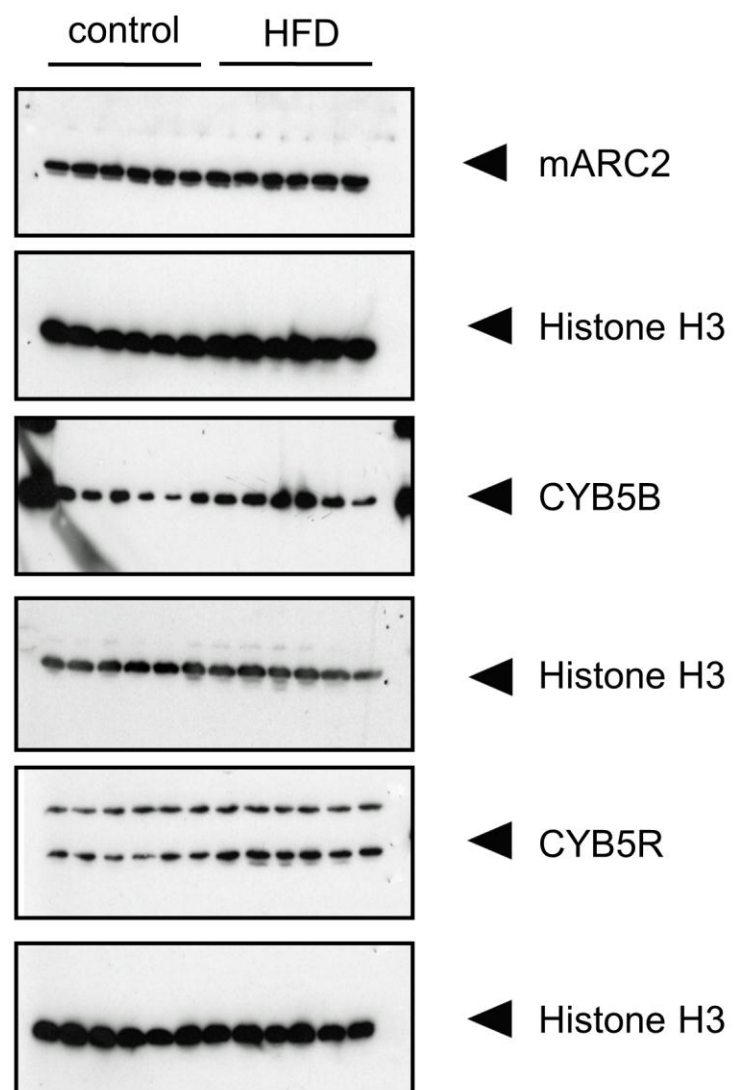**B**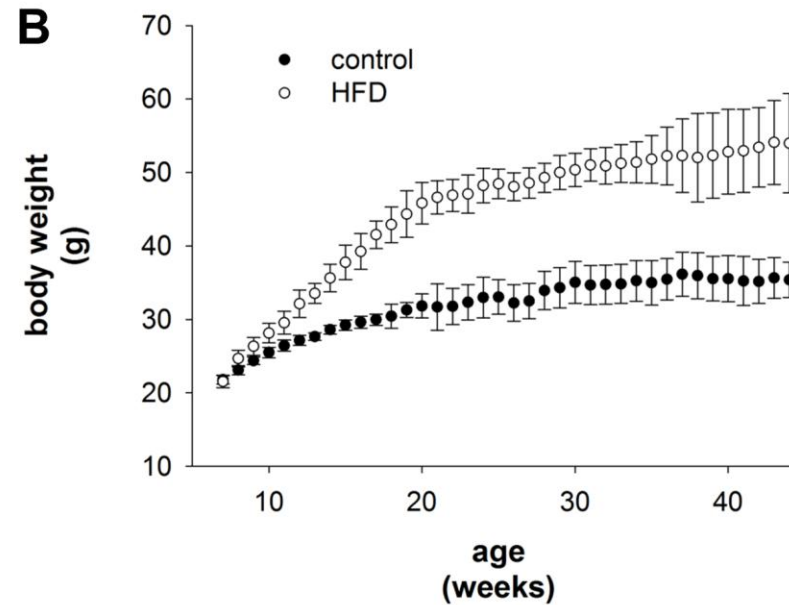**C**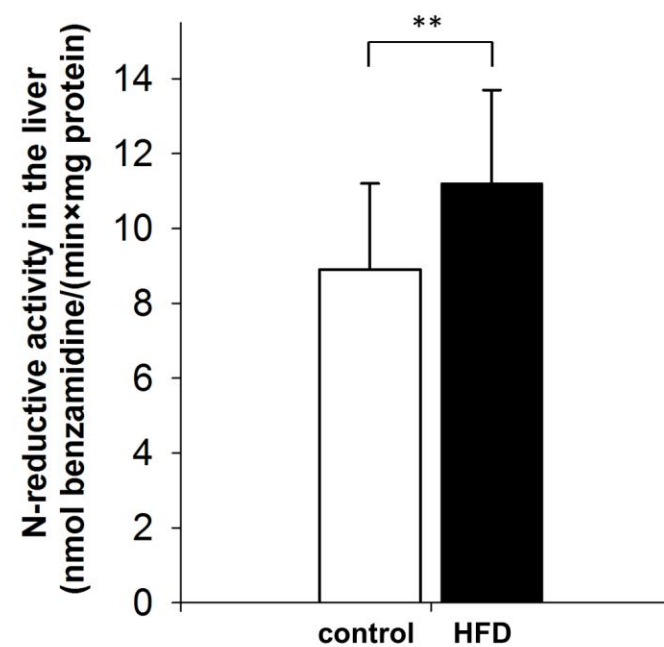

Supplement: Figure S5 — HFD has only minor effects on N -reductive complex abundance and activity in older mice. One group of C57BL/6W mice was fed with regular, another with HFD chow; mice were sacrificed at 48 weeks of age and livers collected. A Protein levels of mARC2, CYB5B and CYB5R in liver homogenates examined by Western Blot, histone H3 was used as loading control. B Development of bodyweight during aging of mice. C N-reductive activity determined by the reduction of model compound benzamidoxime in liver homogenate. The resulting metabolite benzamidine was quantified by HPLC analysis. Determined activities are means ± SD of 5–7 biological samples, each measured as duplicates. Statistical significance was assessed by the U-test. p-values <0.05 were considered significant (**) = p<0,001. (PDF) [file pone.0105371.s005.pdf]
